# C57BL/6J, DBA/2J, and DBA/2J*.Gpnmb^+^* mice have different visual signal processing in the inner retina

**Published:** 2010-12-31

**Authors:** Vittorio Porciatti, Tsung-Han Chou, William J. Feuer

**Affiliations:** Bascom Palmer Eye Institute, University of Miami Miller School of Medicine, Miami, FL

## Abstract

**Purpose:**

To characterize differences in retinal ganglion cell (RGC) function in mouse strains relevant to disease models. C57BL/6J (B6) and DBA/2J (D2) are the two most common mouse strains; D2 has two mutated genes, tyrosinase-related protein 1 (*Tyrp1*) and glycoprotein non-metastatic melanoma protein B (*Gpnmb*), causing iris disease and intraocular pressure (IOP) elevation after 6 months of age that results in RGC degeneration, and is the most widely used model of glaucoma. DBA/2J.*Gpnmb^+^* (D2.*Gpnmb^+^*) is the wild type for the *Gpnmb* mutation and does not develop IOP elevation and glaucoma.

**Methods:**

Young (2–4 months of age) B6, D2, and D2.*Gpnmb^+^* mice (n=6 for each group) were tested with pattern electroretinogram (PERG) in response to different contrasts and spatial frequencies. PERG amplitude and latency dependencies on stimulus parameters (transfer functions) were established for each mouse strain, together with corresponding thresholds for contrast and spatial resolution.

**Results:**

PERG analysis showed that B6, D2, and D2.*Gpnmb^+^* mice had comparable contrast threshold and spatial resolution. Suprathreshold spatial contrast processing, however, had different characteristics in the three strains. PERG amplitude and latency changes with increasing contrast were different between B6 and D2 as well as between D2 and D2*.Gpnmb^+^.*

**Conclusions:**

B6, D2, and D2.*Gpnmb^+^* mice have different characteristics of PERG spatial contrast processing consistent with different mechanisms of contrast gain control. This may imply differences in the activity of underlying PERG generators and synaptic circuitry in the inner retina.

## Introduction

The two most common inbred mouse strains C57BL/6J (B6) and DBA/2J (D2) differ in several specific functions. These include differential sensitivity to nociceptive stimuli [1], taste [2], alcohol, barbiturates, and cocaine [3,4]. Visual behaviors, such as visual detection, pattern discrimination, and visual acuity, are reported to be similar in young (within 4 months of age) B6 and D2 mice [5]. The electroretinogram (ERG) is also reported to be similar in young B6 and D2 mice [6,7]. However, retinal ganglion cell (RGC) population is reported to be significantly larger in D2 mice than in B6 mice [8]. It is possible that there are differences in RGC function between B6 and D2 strains that are not reflected in measures of either visual behavior or ERG and that probe primarily the preganglionic retinal activity [9]. As mouse models of RGC death, glaucoma, and optic neuropathy using B6 and D2 genetic backgrounds are increasingly used [10-12], we wanted to determine if there is a basic difference in RGC function between the two control B6 and D2 strains. We also wanted to determine if there is a difference between the most widely used D2 mouse model of intraocular pressure (IOP) elevation and glaucoma [13-15] and its control DBA/2J.*Gpnmb^+^*, which does not develop glaucoma [16], at ages before the development of high IOP in D2.

We used the pattern electroretinogram (PERG) to systematically investigate the physiologic characteristics of RGC response in 2–4-month-old mice. There is a large body of evidence that PERG reflects RGC electrical activity in mammals [9,17], including mice [18-21]. The PERG is currently used to probe abnormalities of RGC function in mouse models of glaucoma [22,23] and optic nerve disease [24,25].

Results show that the PERG spatial contrast gain control characteristics differ between B6 and D2 mice. PERG spatial contrast gain control characteristics also differ between D2 and D2.*Gpnmb^+^* mice. Altogether, results suggest that neural processing involving RGC differs among these genotypes. Preliminary results of this study have been previously published in abstract form [26].

## Methods

### Animals and husbandry

All procedures were performed in compliance with the Association for Research in Vision and Ophthalmology (ARVO) statement for use of animals in ophthalmic and vision research. The experimental protocol was approved by the Animal Care and Use Committee of the University of Miami. A total of 18 mice (B6, n=6; D2, n=6; D2.*Gpnmb^+^*, n=6; Jackson Labs, Bar Harbor, ME) were tested in the age range 2 to 4 months. Mice were maintained in a cyclic light environment (12 h:12 h light [50 lux]–dark) and fed ad libitum.

### Pattern electroretinogram recording

Detailed description of the PERG technique is reported elsewhere [19,20,27]. In brief, mice were weighed and anesthetized with intraperitoneal injections (0.5–0.7 ml/kg) of a mixture of ketamine (42.8 mg/ml) and xylazine (8.6 mg/ml). Mice were then gently restrained in a custom-made holder that allowed unobstructed vision. The body of the animal was kept at a constant body temperature of 37.0 °C using a feedback-controlled heating pad (TCAT-2LV; Physitemp Instruments, Inc. Clifton, NJ).

A PERG electrode (0.25 mm diameter silver wire-World Precision Instruments, Sarasota, FL-configured to a semicircular loop of 2 mm radius) was placed on the extrapupillary corneal surface by means of a micromanipulator. A small drop of balanced saline was topically applied every 30 min to prevent corneal dryness. Reference and ground electrodes were stainless steel needles (Grass, West Warwick, RI) inserted under the skin and scalp (reference) and tail (ground).

Visual stimuli consisted of contrast-reversing (1 Hz, 2 reversals) horizontal bars generated by a programmable graphic card (VSG-; Cambridge Research Systems, Rochester, UK) on a cathode-ray tube (CRT) display (Sony Multiscan 500, Sony Electronics Inc., San Diego, CA) with the center aligned with the projection of the pupil. The pupils were not dilated, and eyes were not refracted for the viewing distance since the mouse eye has a large depth of focus [28-30]. At the viewing distance of 15 cm, the stimulus field covered an area of 69.4×63.4°. Patterns had fixed mean luminance of 50 cd/m^2^ and variable contrast (0.1 to 1 in ten steps) and spatial frequency (0.05 to 0.8 cycles/degree in five steps). The luminance of the CRT display was γ-corrected using a photometer (OptiCal OP200-E; Cambridge Research Systems Ltd., Rochester, UK). Contrast was defined as C=(Lmax–Lmin)/(Lmax+Lmin), where Lmax=luminance of the bright stripes and Lmin=luminance of the dark stripes [31].

**Figure 1:**
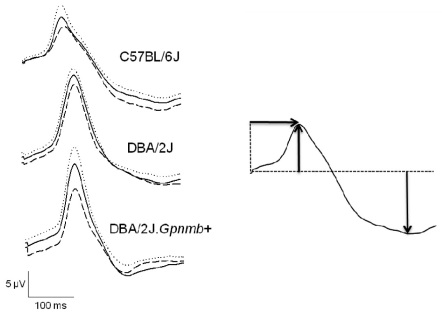
Pattern electroretinogram (PERG) phenotype in C57BL/6J and DBA/2J mice. Grand-average waveforms of maximal pattern electroretinograms recorded in different mouse strains at 2–4 months of age (n=6 for each strain) in response to reversing gratings (temporal frequency 1 Hz, spatial frequency 0.05 cycles/deg, contrast 1.0). For all waveforms, the continuous black line represents the grand average, and the superimposed dotted and dashed lines represent the ±standard error of the mean. In the idealized waveform (right panel), the upward arrow represents the amplitude of the positive peak with latency of 90–120 ms (P100), the downward arrow represents the amplitude of the negative trough with latency of 200–300 ms (N 250), and the horizontal arrow represents the implicit time of the P100 component.

Three consecutive PERG responses to 600 contrast reversals each were recorded. The responses were superimposed to check for consistency and then averaged (1,800 sweeps). The waveform of averaged PERGs to high-contrast (1.0) gratings of low spatial frequency (0.05 cycles/deg) consisted of a major positive peak at around 90–120 ms (defined as P100) followed by a slower negative wave with a broad trough at around 200–300 ms (defined as N250, examples in Figure 1). Note that the human transient PERG also consists of a positive–negative complex. However, the human positive wave peaks at about 50 ms (P50), and the trough of the subsequent negative wave occurs at about 95 ms (N95). It is commonly thought that the N95 wave is more specifically related to RGC function and is more affected than the P50 wave in optic nerve disease [32]. In contrast, the P50 wave is thought to have a preganglionic origin and be affected in macular diseases [32]. In the mouse transient PERG, the positive (P100) and negative components (N250) do not appear to dissociate in disease models; both the P100 and the N250 components are altered in glaucoma [19] as well as after selective RGC degeneration induced by optic nerve crush [21]. The PERG responses represented in Figure 1 were obtained under conditions that maximize response amplitude (0.05 cycles/deg, 1.0 contrast), thereby yielding a robust response—defined here as maximal PERG—that has been used in several studies on mouse models of optic neuropathies [19,22-24,33-35]. Both P100 and N250 components were evaluated.

In the present study, the entire dynamic range of the PERG response to spatial contrast was investigated. As the level of PERG signal progressively decreased with decreasing contrast and increasing spatial frequency, manual identification of P100 and N250 components would have potentially introduced operator bias in waveforms close to response threshold. To prevent this, maximal voltage in the expected time window for P100 (50–200 ms) and minimal voltage in the expected time window for N250 (201–350 ms) were automatically identified using a simple macro written in Sigmaplot language (version 11.2; Systat Software, Inc., San Jose, CA). For the analysis of contrast transfer function and spatial transfer function, response amplitude was defined as the peak-to-trough voltage (P100–N250); response latency was defined as the time-to-peak of the P100 wave. The latency of the N250 component was not systematically investigated since, in many instances, was rather broad, precluding accurate peak-time measurement of this component. The time-to-peak of the negative trough (N95) of the human transient PERG is not currently evaluated for the same reason [36].

### Statistical analysis

For statistical analysis, responses of the two eyes were averaged and used as a single entry. Strain differences in absolute amplitude and latency of P100 and N250 components of maximal PERG (0.05 cycles/deg, 1.0 contrast) were analyzed with Students *t-* tests. To compare transfer functions, peak-to-trough (P100-N250) response amplitudes and P100 latencies were first normalized to the maximal PERG. The normalized PERGs of B6 and D2.*Gpnmb^+^* mouse strains to different contrast- and spatial frequency stimuli were then each compared to D2 mice with a two-factor subject (mouse strain) by repeated measures (stimulus levels) analysis of variance (ANOVA) with orthogonal polynomial decomposition, followed by post hoc *t* tests. A p value of <0.05 was considered significant.

## Results

### Comparison between B6 and D2 strains: maximal PERG response

**Figure 2:**
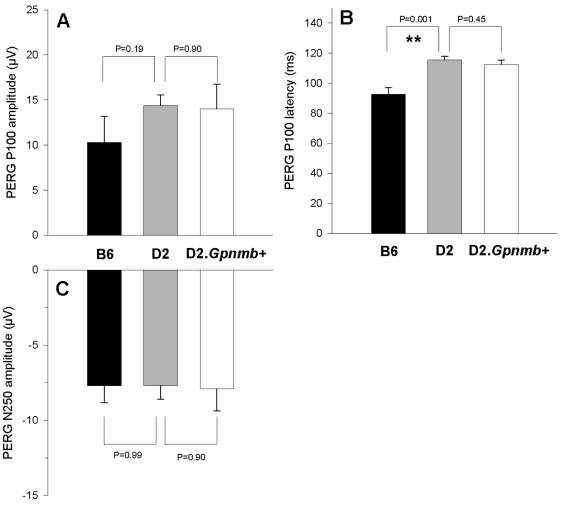
Analysis of maximal pattern electroretinogram (PERG) components in different mouse strains (n=6 for each group). Data have been obtained from measurements of individual waveforms in response to 1 Hz reversing gratings (spatial frequency 0.05 cycles/degree, contrast 1.0). **A**: Mean amplitude of the positive peak with latency around 100 ms (P100) component. **B**: Mean amplitude of the negative trough with latency around 250 ms (N250) component. **C**: Mean latency of the P100 component. In all panels, the error bars represent the standard error of the mean. Brackets superimposed to adjacent bars represent statistical comparisons (p value, *t* test) between means of C57BL/6J (B6) and DBA/2J (D2) and between means of DBA/2J (D2) and DBA/2J.*Gpnmb^+^*. The level of statistical significance is also marked with one asterisk (*) if p<0.05 and two (**) if p<0.01.

Examples of maximal PERGs in response to contrast reversal gratings (temporal frequency=1Hz, spatial frequency=0.05 cycles/deg, contrast=1.0) for the three mouse strains are displayed in Figure 1 as group averages±standard error of the mean. It is apparent in Figure 1 that in B6 mice the PERG tended to have a shorter latency compared to both D2 and D2.*Gpnmb^+^*, whereas waveforms were similar in D2 strains. Evaluation of P100 and N250 components was performed on individual waveforms and their mean displayed in Figure 2. The amplitude of the P100 component tended to be smaller in B6 than in D2, but the difference was not significant (*t* test, p=0.19). The P100 component had a similar amplitude in D2 and D2.*Gpnmb^+^*. The N250 component had virtually identical amplitude in all strains. On average, the latency of the PERG P100 component was substantially shorter in B6 mice than in D2 mice by about 22.7 ms (*t* test, p=0.001), whereas the latency of D2 and D2.*Gpnmb^+^* was similar.

### PERG contrast response function: comparison between B6 and D2 strains

**Figure 3:**
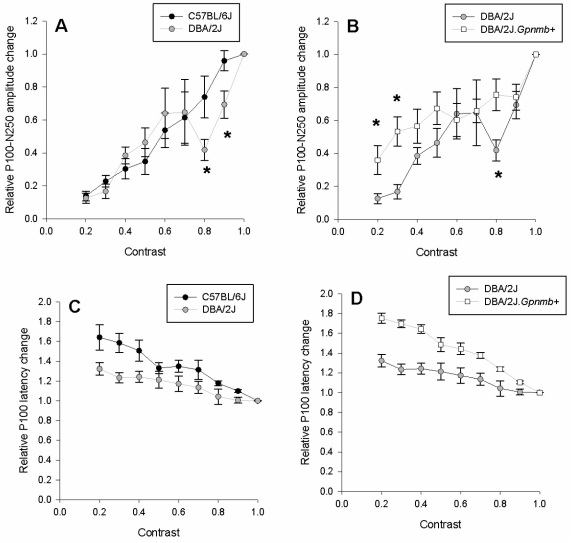
Contrast transfer function of pattern electroretinogram (PERG) amplitude (**A**, **B**) and latency (**C**, **D**) for different mouse strains. All responses have been obtained at a fixed spatial frequency of 0.05 cycles/deg and temporal frequency of 1 Hz. In all panels, symbols represent the mean±standard error of the mean (n=6 mice for each strain). Amplitude and latency changes are expressed in relative units compared to the maximal PERG in response to gratings of 0.05 cycles/degree and contrast of 1.0 reversing at 1 Hz, corresponding waveforms of which are shown in Figure 1.

Figure 3 shows how the PERG amplitude and latency change as a function of stimulus contrast for a fixed spatial frequency of 0.05 cycles/degree. To appreciate differences in the function among strains, all data were expressed as relative changes compared to the maximal PERG, waveforms and absolute values of which are shown in Figure 1 and Figure 2. With decreasing contrast, the PERG amplitude progressively decreased while the latency progressively increased in all strains. However, there were notable differences among strains. As shown in Figure 3A, in B6 mice the contrast function of amplitude was approximately linear over the entire contrast range, whereas in D2 the contrast function had a more complex shape. In particular, the function was approximately linear between 0.2 and 0.6 contrast, displayed a local minimum (notch) at 0.8 contrast, and a second linear branch at 0.8–1.0 contrasts. The response latency (Figure 3C) increased approximately linearly with decreasing contrast in both B6 and D2. The slope of latency increase with decreasing contrast tended to be steeper in B6 compared to D2. At contrast of 0.1, PERG responses of both B6 and D2 were indistinguishable from a control response obtained with the stimulus occluded (noise) and were not included in the figure. We considered the contrast threshold being located at some point between contrasts of 0.1 and 0.2.

### PERG contrast response function: comparison between D2 and D2.*Gpnmb*^+^ strains

As shown in Figure 3B, the form of the contrast amplitude appears to be different between D2 and D2.*Gpnmb^+^* mice. In particular, the amplitude notch at 0.8 contrast visible in D2 mice was not present in D2.*Gpnmb^+^* mice. At low (0.2–0.3) contrast the PERG amplitude was relatively higher in D2.*Gpnmb^+^* mice compared to D2. The response latency (Figure 3D) increased approximately linearly with decreasing contrast in both D2 and D2.*Gpnmb^+^* mice. The slope of latency increase with decreasing contrast tended to be steeper in B6 compared to D2. At contrast of 0.1, PERG responses of both D2 and D2.*Gpnmb^+^* mice were indistinguishable from a control response obtained with the stimulus occluded (noise) and were not included in the figure. We considered the contrast threshold being located at some point between 0.1 and 0.2 contrast.

### Statistical comparisons among strains

#### B6 versus D2

There was a statistically significant strain by contrast level interaction in normalized PERG amplitude means (p=0.041, repeated measures ANOVA; see panel A). Post-hoc *t*-tests revealed no significant differences between strains for contrast levels 0.2 thru 0.6; however, there were significant differences between strains for contrast levels 0.8 (p=0.050) and 0.9 (p=0.029). Latency became significantly greater with decreasing contrast in B6 mice compared to D2 mice (p=0.015, panel C), while no interaction was observed (p=0.25). In summary, the form of PERG amplitude contrast function significantly differed between B6 and D2 strains at high contrasts. PERG latency significantly differed between B6 and D2 strains, but the form of the latency transfer function was similar.

#### D2 versus D2.Gpnmb^+^

There was a statistically significant strain by contrast level interaction in normalized PERG amplitude means (p=0.031, repeated measures ANOVA; see panel B). Post hoc *t* tests revealed differences that were significant at contrast levels 0.2 (p=0.031), 0.3 (p=0.004), and 0.8 (p=0.015). There was a highly significant strain by contrast level interaction in normalized PERG latency means (p=0.001, repeated measures ANOVA; see panel D). Post hoc *t* tests on PERG latency could have been performed; however, orthogonal polynomial decomposition revealed that the interaction was due to differences in the slopes of the linear relation of PERG response to contrast between the two strains of mice (p=0.001). In summary, the form of PERG amplitude contrast function significantly differed between D2 and D2.*Gpnmb^+^* strains at both low and high contrasts. PERG latency significantly differed between the two strains, but the form of the latency transfer function was similar.

**Figure 4:**
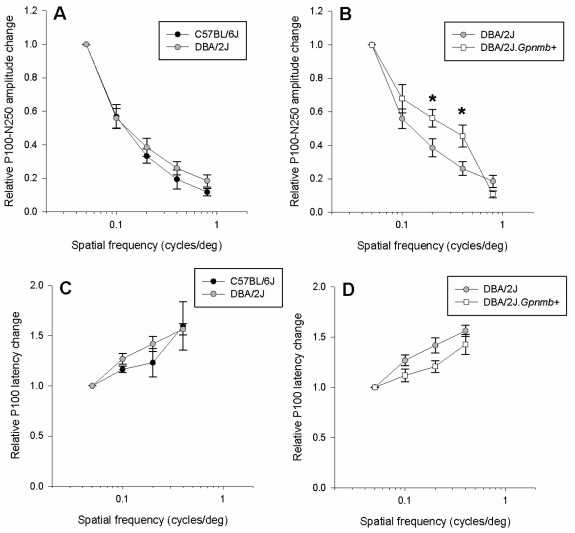
Spatial transfer function of pattern electroretinogram (PERG) amplitude (**A**, **B**) and latency (**C**, **D**) for different mouse strains. All responses have been obtained at a fixed temporal frequency of 1 Hz and contrast of 1.0. In all panels, symbols represent the mean±standard error of the mean. Amplitude and latency changes are expressed in relative units compared to the maximal PERG in response to gratings of 0.05 cycles/degree and contrast of 1.0 reversing at 1 Hz, corresponding waveforms of which are shown in Figure 1.

Figure 4 shows how the PERG amplitude and latency change as a function of spatial frequency (range 0.05–0.8 cycles/deg) for a fixed temporal frequency of 1 Hz and contrast of 1. As for the contrast functions shown above, all data were expressed as relative changes compared to the maximal PERG, waveforms and absolute values of which are shown in Figure 1 and Figure 2. With increasing spatial frequency, the PERG amplitude progressively decreased, while latency increased, in all strains. At 0.8 cycles/degree, the PERG amplitude was just above the noise level (the amplitude of a response with the stimulus occluded) in all strains. We considered this spatial frequency as an index of retinal visual acuity. The PERG latency at 0.8 cycles/degree was not included in the figure since at this spatial frequency the signal was very close to the noise level and the automatic peak evaluation produced unreliable estimates.

Statistical comparisons were performed with the same approach used for the contrast function shown in Figure 3. That is, the normalized PERG means of B6 and D2.*Gpnmb^+^* mouse strains to different spatial frequencies were each compared to D2 mice with a two-factor subject (mouse strain) by repeated measures (contrast level) ANOVA.

#### B6 versus D2

There was no statistically significant difference in PERG amplitude between strains (p=0.26) and strain by spatial frequency interaction (p=0.75, panel A). There was no statistically significant difference in PERG latency between strains (p=0.37) and strain by spatial frequency interaction (p=0.55, panel C). In summary, there were no significant differences in the form of both amplitude and latency spatial functions between B6 and D2 strains.

#### D2 versus D2.Gpnmb^+^

There was a statistically strain by spatial frequency interaction (p=0.011, panel B) in PERG amplitude. Post hoc *t* tests revealed significant strain differences at 0.2 cycles/deg (p=0.032) and at 0.4 cycles/deg (p=0.028). There was a statistically significant difference in PERG latency between strains (p=0.001) but no strain by spatial frequency interaction (p=0.89, panel D). In summary, there was a significant difference in the form of amplitude function between D2 and D2.*Gpnmb^+^* strains for intermediate spatial frequencies. PERG latency significantly differed between the two strains, but the form of the latency transfer function was similar.

## Discussion

The PERG is a specialized kind of ERG that reflects inner activity and it represents an effective tool to assess normal and abnormal RGC function. In mouse models of optic nerve degeneration, the PERG may help to understand how genetic diversity relates to specific differences in RGC function and susceptibility to stress [15]. In this study we have used the PERG to characterize the spatial contrast properties of RGC response in the two most common inbred mouse strains, C57BL/6J (B6) and DBA/2J (D2), which are used in several disease models. We also tested a relevant DBA/2J substrain, D2.*Gpnmb^+^* that has a wild-type glycoprotein non-metastatic melanoma protein B (*Gpnmb*) allele but no other known differences to modern D2 mice [37].

Our results show that the PERGs of B6, D2, and D2.*Gpnmb^+^* displayed many similarities but some notable differences. The waveform of PERG obtained under conditions that maximize the signal (spatial frequency 0.05 cycles/degree, max contrast 1.0) [19] differed between B6 and D2 strains. In D2 strains the PERG had a substantially longer latency (about 20 ms) compared to B6. No obvious differences in amplitude and latency between D2 and D2.*Gpnmb^+^* strains were observable. The major difference among the three mouse strains was the way PERG amplitude and latency changed as a function of spatial contrast (contrast transfer function). In B6 mice, the contrast transfer function of PERG amplitude was linear, whereas in D2 there was a clear notch in PERG amplitude at a contrasts of 0.8. In addition, the slope of latency increase associated with decreasing contrast was shallower in D2 compared to B6. The amplitude notch at 0.8 contrast occurring in D2 mice was not present in D2.*Gpnmb^+^* mice, which had a more robust response at lower contrasts. In addition, the slope of latency increase associated with decreasing contrast was shallower in D2 compared to D2.*Gpnmb^+^* mice. Amplitude notches at high contrast have been reported before for the visually evoked potentials (VEP) [38,39], but their origin is still a matter of speculation. One possibility is that the notch originates from the interaction between different underlying neural generators that respond with different latency, resulting in amplitude cancellation.

Overall, differences in contrast transfer functions of amplitude and latencies can be understood in terms of different mechanisms of contrast gain control in the PERG generators [40]. Photoreceptors do not adapt to contrast, whereas RGC typically display substantial gain control [41]. Contrast gain control is a mechanism whereby RGC adjust their responsiveness (both in terms of amplitude and latency) through feedback conductances, thereby allowing more efficient use of their dynamic range. Contrast gain control mechanisms are expected to play a major role at sites where there is a large convergence of neural inputs to a target neuron [41]. As in the mouse retina there is a large convergence between photoreceptors and RGC, this might explain the remarkable changes of the PERG signal latency with changing contrast that we found. Altogether, our results suggest that neural processing in the inner retina for suprathreshold contrast stimuli differs between B6 and D2 mice. D2 and D2.*Gpnmb^+^* also displayed differences for suprathreshold contrast stimuli. At threshold contrasts, however, contrast gain control mechanisms are expected to play a lesser role. In all strains, the PERG contrast threshold was very similar, in the order of 10%–20%. This value is in keeping with previous reports in B6 mice obtained with PERG [18], VEP [42], and optomotor [43-45] studies but somewhat lower than that obtained with optokinetic response [46,47] and intrinsic optical imaging [48].

The spatial frequency function of PERG amplitude (obtained at maximum contrast) was similar in B6 and D2 mice but displayed subtle differences between D2 and D2.*Gpnmb^+^*. The spatial frequency threshold (acuity) was about 0.8 cycles/degree in all strains. This value is in keeping with previous reports on visual acuity in B6 mice obtained with PERG [18,49], VEP [42,49-51], and behavior [52] but somewhat higher than that reported for optomotor response [45,53], intrinsic optical imaging [48], and swim tasks [5].

With increasing spatial frequency, the PERG latency increased dramatically over the spatial frequency range in all strains. This space–time association lends further support to the notion that considerable convergence and spatial summation is at play in inner retinal circuitry [54,55]. Convergence and spatial summation are mechanisms whereby more synapses are simultaneously activated with increasing stimulus size (decreasing spatial frequency), leading to a larger compound synaptic potential that reaches threshold faster [56].

RGC population in DBA/2J mice is reported to be significantly larger (63,351±1208) than that of C57BL/6J (54,630±874) [8]. This may have a counterpart in a different inner retina circuitry between B6 and D2 mice resulting in different spatial contrast functions. Differences in the PERG spatial contrast function between D2 and D2.*Gpnmb^+^* mice, however, are likely to result from factors other than RGC number. D2 mice have mutations in two genes, the tyrosinase-related protein 1 (*Tyrp1,* which is linked to iris stromal atrophy) and the transmembrane glycoprotein *nmb* (*Gpnmb^R150X^*, which is linked to iris pigment dispersion) [13,57]. The function(s) of the *Gpnmb* gene are not well known. *Gpnmb* influences the glaucoma phenotype of D2 mice [37]. D2 mice wild type for the *Gpnmb^R150X^* mutation (D2. *Gpnmb^+^*) develop mild iris disease and modest IOP elevation but not glaucomatous nerve damage [16]. Low levels of GPNMB protein are also expressed in the neuronal retina of DBA/2J mice [37] and monkeys [58]. Differential expression of GPNMB in the inner retina of D2 and D2.*Gpnmb^+^* mice may have a counterpart in a different RGC function.

In summary, PERG analysis shows that B6, D2, and D2.*Gpnmb^+^* mice have comparable thresholds for contrast and spatial frequency. Suprathreshold spatial contrast processing, however, has different characteristics in these mouse strains, implying different synaptic circuitry in the inner retina. It remains to be established whether these differences have a counterpart in susceptibility to RGC to insult or disease.

## References

[1] Mogil JS Richards SP O'Toole LA Helms ML Mitchell SR Belknap JK Genetic sensitivity to hot-plate nociception in DBA/2J and C57BL/6J inbred mouse strains: possible sex-specific mediation by delta2-opioid receptors. Pain 1997 70 267 77 9150302 10.1016/s0304-3959(97)03333-2

[2] Boughter JD Raghow S Nelson T Munger S Inbred mouse strains C57BL/6J and DBA/2J vary in sensitivity to a subset of bitter stimuli. BMC Genet 2005 6 36 15967025 10.1186/1471-2156-6-36 PMC1183203

[3] Belknap JK Deutsch CK Differential neurosensitivity to three alcohols and phenobarbital in C57BL/6J and DBA/2J mice. Behav Genet 1982 12 309 17 7126108 10.1007/BF01067850

[4] Belknap JK Noordewier B Lame M Genetic dissociation of multiple morphine effects among C57BL/6J, DBA/2J and C3H/HeJ inbred mouse strains. Physiol Behav 1989 46 69 74 2813556 10.1016/0031-9384(89)90324-7

[5] Wong AA Brown RE Visual detection, pattern discrimination and visual acuity in 14 strains of mice. Genes Brain Behav 2006 5 389 403 16879633 10.1111/j.1601-183X.2005.00173.x

[6] Pinto LH Invergo B Shimomura K Takahashi JS Troy JB Interpretation of the mouse electroretinogram. Doc Ophthalmol 2007 115 127 36 17636411 10.1007/s10633-007-9064-y PMC3786689

[7] Bayer AU Neuhardt T May AC Martus P Maag KP Brodie S Lutjen-Drecoll E Podos SM Mittag T Retinal morphology and ERG response in the DBA/2NNia mouse model of angle-closure glaucoma. Invest Ophthalmol Vis Sci 2001 42 1258 65 11328737

[8] Williams RW Strom RC Rice DS Goldowitz D Genetic and environmental control of variation in retinal ganglion cell number in mice. J Neurosci 1996 16 7193 205 8929428 10.1523/JNEUROSCI.16-22-07193.1996 PMC6578945

[9] Zrenner E. The physiological basis of the pattern electroretinogram. In: Osborne N, Chader G, editors. Progress in Retinal Research 1990;9;427–64.

[10] McKinnon SJ Schlamp CL Nickells RW Mouse models of retinal ganglion cell death and glaucoma. Exp Eye Res 2009 88 816 24 19105954 10.1016/j.exer.2008.12.002 PMC3056071

[11] Pang IH Clark AF Rodent models for glaucoma retinopathy and optic neuropathy. J Glaucoma 2007 16 483 505 17700292 10.1097/IJG.0b013e3181405d4f

[12] Johnson TV Tomarev SI Rodent models of glaucoma. Brain Res Bull 2010 81 349 58 19379796 10.1016/j.brainresbull.2009.04.004 PMC2830899

[13] Chang B Smith RS Hawes NL Anderson MG Zabaleta A Savinova O Roderick TH Heckenlively JR Davisson MT John SW Interacting loci cause severe iris atrophy and glaucoma in DBA/2J mice. Nat Genet 1999 21 405 9 10192392 10.1038/7741

[14] Libby RT Gould D Anderson M John S Complex genetics of glaucoma susceptibility. Annu Rev Genomics Hum Genet 2005 6 15 44 16124852 10.1146/annurev.genom.6.080604.162209

[15] Howell GR Libby RT John SW Mouse genetic models: an ideal system for understanding glaucomatous neurodegeneration and neuroprotection. Prog Brain Res 2008 173 303 21 18929118 10.1016/S0079-6123(08)01122-9

[16] Howell GR Libby RT Marchant JK Wilson LA Cosma IM Smith RS Anderson MG John SW Absence of glaucoma in DBA/2J mice homozygous for wild-type versions of Gpnmb and Tyrp1. BMC Genet 2007 8 45 17608931 10.1186/1471-2156-8-45 PMC1937007

[17] Mafei L Fiorentini A Electroretinographic responses to alternating gratings before and after section of the optic nerve. Science 1981 211 953 5 7466369 10.1126/science.7466369

[18] Porciatti V Pizzorusso T Cenni MC Maffei L The visual response of retinal ganglion cells is not altered by optic nerve transection in transgenic mice overexpressing Bcl-2. Proc Natl Acad Sci USA 1996 93 14955 9 8962163 10.1073/pnas.93.25.14955 PMC26244

[19] Porciatti V Saleh M Nagaraju M The pattern electroretinogram as a tool to monitor progressive retinal ganglion cell dysfunction in the DBA/2J mouse model of glaucoma. Invest Ophthalmol Vis Sci 2007 48 745 51 17251473 10.1167/iovs.06-0733 PMC1794678

[20] Porciatti V The mouse pattern electroretinogram. Doc Ophthalmol 2007 115 145 53 17522779 10.1007/s10633-007-9059-8 PMC2773675

[21] Miura G Wang MH Ivers KM Frishman LJ Retinal pathway origins of the pattern ERG of the mouse. Exp Eye Res 2009 89 49 62 19250935 10.1016/j.exer.2009.02.009 PMC2739005

[22] Saleh M Nagaraju M Porciatti V Longitudinal Evaluation of Retinal Ganglion Cell Function and IOP in the DBA/2J Mouse Model of Glaucoma. Invest Ophthalmol Vis Sci 2007 48 4564 72 17898279 10.1167/iovs.07-0483 PMC2765717

[23] Howell GR Libby RT Jakobs TC Smith RS Phalan FC Barter JW Barbay JM Marchant JK M. N, Porciatti V, Whitmore AV, Masland RH, John SW. Axons of retinal ganglion cells are insulted in the optic nerve early in DBA/2J glaucoma. J Cell Biol 2007 179 1523 37 18158332 10.1083/jcb.200706181 PMC2373494

[24] Guy J Qi X Koilkonda RD Arguello T Chou TH Ruggeri M Porciatti V Lewin AS Hauswirth WW Efficiency and safety of AAV-mediated gene delivery of the human ND4 complex I subunit in the mouse visual system. Invest Ophthalmol Vis Sci 2009 50 4205 14 19387075 10.1167/iovs.08-3214 PMC3085487

[25] Koilkonda RD Chou T-H Porciatti V Hauswirth WW Guy J Self-Complementary AAV Induces Rapid and Highly Efficient Allotopic Expression of the Human ND4 Complex I Subunit in the Mouse Visual System. Invest Ophthalmol Vis Sci 2010 51 4494

[26] Chou T-H Nagaraju M Porciatti V The PERG Phenotype of DBA/2J Mice Compared to C57BL/6J Mice. Invest Ophthalmol Vis Sci 2008 49 719

[27] Porciatti V Nagaraju M Head-up tilt lowers IOP and improves RGC dysfunction in glaucomatous DBA/2J mice. Exp Eye Res 2010 90 452 60 20036238 10.1016/j.exer.2009.12.005 PMC2824077

[28] Remtulla S Hallett PE A schematic eye for the mouse, and comparisons with the rat. Vision Res 1985 25 21 31 3984214 10.1016/0042-6989(85)90076-8

[29] Schmucker C Schaeffel F A paraxial schematic eye model for the growing C57BL/6 mouse. Vision Res 2004 44 1857 67 15145680 10.1016/j.visres.2004.03.011

[30] Artal P Herreros de Tejada P Munoz Tedo C Green DG Retinal image quality in the rodent eye. Vis Neurosci 1998 15 597 605 9682864 10.1017/s0952523898154020

[31] Michelson A. Studies in optics. Chicago: University of Chicago Press; 1927.

[32] Holder GE Pattern electroretinography (PERG) and an integrated approach to visual pathway diagnosis. Prog Retin Eye Res 2001 20 531 61 11390258 10.1016/s1350-9462(00)00030-6

[33] Nagaraju M Saleh M Porciatti V IOP-Dependent Retinal Ganglion Cell Dysfunction in Glaucomatous DBA/2J Mice. Invest Ophthalmol Vis Sci 2007 48 4573 9 17898280 10.1167/iovs.07-0582 PMC2031015

[34] Chou T-H Borja D Kocaoglu OP Uhlhorn SR Manns F Porciatti V Postnatal Growth of Eye Size in DBA/2J Mice Compared With C57BL/6J Mice: In-vivo Analysis With OCT. Invest Ophthalmol Vis Sci 2009 50 2776 10.1167/iovs.10-6340 PMC3109044 21372015

[35] Koilkonda RD Chou TH Porciatti V Hauswirth WW Guy J Induction of rapid and highly efficient expression of the human ND4 complex I subunit in the mouse visual system by self-complementary adeno-associated virus. Arch Ophthalmol 2010 128 876 83 20625049 10.1001/archophthalmol.2010.135 PMC3431796

[36] Holder GE Brigell MG Hawlina M Meigen T Vaegan, Bach M. ISCEV standard for clinical pattern electroretinography-2007 update. Doc Ophthalmol 2007 114 111 6 17435967 10.1007/s10633-007-9053-1 PMC1896293

[37] Anderson MG Nair KS Amonoo LA Mehalow A Trantow CM Masli S John SW GpnmbR150X allele must be present in bone marrow derived cells to mediate DBA/2J glaucoma. BMC Genet 2008 9 30 18402690 10.1186/1471-2156-9-30 PMC2373794

[38] Nakayama K Mackeben M Steady state visual evoked potentials in the alert primate. Vision Res 1982 22 1261 71 7179746 10.1016/0042-6989(82)90138-9

[39] Strasburger H Scheidler W Rentschler I Amplitude and phase characteristics of the steady-state visual evoked potential. Appl Opt 1988 27 1069 88 20531521 10.1364/AO.27.001069

[40] Shapley RM Victor JD The effect of contrast on the transfer properties of cat retinal ganglion cells. J Physiol 1978 285 275 98 745079 10.1113/jphysiol.1978.sp012571 PMC1281756

[41] Baccus SA Meister M Retina versus Cortex: Contrast Adaptation in Parallel Visual Pathways. Neuron 2004 42 5 7 15066260 10.1016/s0896-6273(04)00187-4

[42] Porciatti V Pizzorusso T Maffei L The visual physiology of the wild type mouse determined with pattern VEPs. Vision Res 1999 39 3071 81 10664805 10.1016/s0042-6989(99)00022-x

[43] Prusky GT Alam NM Beekman S Douglas RM Rapid quantification of adult and developing mouse spatial vision using a virtual optomotor system. Invest Ophthalmol Vis Sci 2004 45 4611 6 15557474 10.1167/iovs.04-0541

[44] Schmucker C Schaeffel F Contrast sensitivity of wildtype mice wearing diffusers or spectacle lenses, and the effect of atropine. Vision Res 2006 46 678 87 15993919 10.1016/j.visres.2005.04.015

[45] Umino Y Solessio E Barlow RB Speed, spatial, and temporal tuning of rod and cone vision in mouse. J Neurosci 2008 28 189 98 18171936 10.1523/JNEUROSCI.3551-07.2008 PMC2847259

[46] Tabata H Shimizu N Wada Y Miura K Kawano K. Initiation of the optokinetic response (OKR) in mice. J Vis 2010 10 13.1 17 20143906 10.1167/10.1.13

[47] van Alphen B Winkelman BHJ Frens MA Age- and Sex-Related Differences in Contrast Sensitivity in C57Bl/6 Mice. Invest Ophthalmol Vis Sci 2009 50 2451 8 19117934 10.1167/iovs.08-2594

[48] Heimel JA Hartman RJ Hermans JM Levelt CN Screening mouse vision with intrinsic signal optical imaging. Eur J Neurosci 2007 25 795 804 17328775 10.1111/j.1460-9568.2007.05333.x

[49] Rossi FM Pizzorusso T Porciatti V Marubio LM Maffei L Changeux JP Requirement of the nicotinic acetylcholine receptor beta 2 subunit for the anatomical and functional development of the visual system. Proc Natl Acad Sci USA 2001 98 6453 8 11344259 10.1073/pnas.101120998 PMC33489

[50] Huang ZJ Kirkwood A Pizzorusso T Porciatti V Morales B Bear MF Maffei L Tonegawa S BDNF regulates the maturation of inhibition and the critical period of plasticity in mouse visual cortex. Cell 1999 98 739 55 10499792 10.1016/s0092-8674(00)81509-3

[51] Ridder WH 3rd Nusinowitz S The visual evoked potential in the mouse–origins and response characteristics. Vision Res 2006 46 902 13 16242750 10.1016/j.visres.2005.09.006

[52] Gianfranceschi L Fiorentini A Maffei L Behavioural visual acuity of wild type and bcl2 transgenic mouse. Vision Res 1999 39 569 74 10341985 10.1016/s0042-6989(98)00169-2

[53] Redfern WS Storey S Tse K Hussain Q Maung KP Valentin JP Ahmed G Bigley A Heathcote D McKay JS Evaluation of a convenient method of assessing rodent visual function in safety pharmacology studies: Effects of sodium iodate on visual acuity and retinal morphology in albino and pigmented rats and mice. J Pharmacol Toxicol Methods 2010 20619348 10.1016/j.vascn.2010.06.008

[54] Sagdullaev BT McCall MA Stimulus size and intensity alter fundamental receptive-field properties of mouse retinal ganglion cells in vivo. Vis Neurosci 2005 22 649 59 16332276 10.1017/S0952523805225142

[55] Weng C Yeh CI Stoelzel CR Alonso JM Receptive field size and response latency are correlated within the cat visual thalamus. J Neurophysiol 2005 93 3537 47 15590731 10.1152/jn.00847.2004

[56] Andreasen M Lambert JD Factors determining the efficacy of distal excitatory synapses in rat hippocampal CA1 pyramidal neurones. J Physiol 1998 507 441 62 9518704 10.1111/j.1469-7793.1998.441bt.x PMC2230798

[57] Anderson MG Smith R Hawes N Zabaleta A Chang B Wiggs J John S Mutations in genes encoding melanosomal proteins cause pigmentary glaucoma in DBA/2J mice. Nat Genet 2002 30 81 5 11743578 10.1038/ng794

[58] Kompass KS Agapova OA Li W Kaufman PL Rasmussen CA Hernandez MR Bioinformatic and statistical analysis of the optic nerve head in a primate model of ocular hypertension. BMC Neurosci 2008 9 93 18822132 10.1186/1471-2202-9-93 PMC2567987

